# Psychometric Validation of the Persian Version of Short Form Self-Regulation Questionnaire in Community-Dwelling Older Adults

**DOI:** 10.3389/fpsyg.2022.844871

**Published:** 2022-06-23

**Authors:** Mohadeseh Motamed-Jahromi, Mohammad Hossein Kaveh, Amin Mohammadpour, Abdolrahim Asadollahi

**Affiliations:** ^1^Student Research Committee, Department of Health Promotion, School of Health, Shiraz University of Medical Sciences, Shiraz, Iran; ^2^Research Center for Health Sciences, Department of Health Promotion, School of Health, Shiraz University of Medical Sciences, Shiraz, Iran; ^3^Department of Environmental Health Engineering, School of Health, Shiraz University of Medical Sciences, Shiraz, Iran; ^4^Department of Health Promotion and Aging, School of Health, Shiraz University of Medical Sciences, Shiraz, Iran

**Keywords:** Persian, self-regulation, validity, psychometric, scale, older adult

## Abstract

The aim of this study was to examine the validity and reliability of the Persian version of the Short Form Self-Regulation Questionnaire (SSRQ) among Iranian community-dwelling older adults and to determine its optimal cutoff point. In Shiraz, Iran, a cross-sectional study of 500 older adults ≥ 60 years was conducted in two steps. The forward–backward method was used for translation. Psychometric properties, such as the face and content validity, based on the point of view of experts, construct validity based on exploratory factor analysis (EFA) and confirmatory factor analysis (CFA), convergent validity by assessing the relationship with the Generalized Self-Efficacy Scale (GSE-10), and reliability based on Cronbach’s α were examined. A receiver operating characteristic curve (ROC) was plotted to confirm the cutoff point. Validity of both the face and the content was confirmed. The first stage of construct validity was performed using the kurtosis test and the EFA, and finally, only 20 items in four subscales were loaded with 76.34% of the total variance. The CFA indicated a good fit to the data (root mean square error of approximation (RMSEA) = 0.059; comparative fit index (CFI) = 0.92; and goodness of fit index (GFI) = 0.89). Cronbach’s α coefficient of the SSRQ-20 increased to 0.87. A significant positive correlation was found between the SSRQ-20 and the GSE-10 (*r* = 0.44), indicating acceptable convergent validity. The optimal cutoff score for differentiating older adults in terms of self-regulation was 71. This study demonstrates that the Persian version of the SSRQ, which contains 20 items, is a valid and reliable tool for assessing self-regulation in Iranian community-dwelling older adults.

## Introduction

The progressive aging of our society is a significant issue in this era. Aging is a natural process that may be experienced by people at different times; however, the World Health Organization (WHO) defines aging as being over 60 years of age (World Health Organization [WHO], 2007). According to the WHO, there are currently 600 million older people worldwide, which is projected to double by 2025 and reach 2 billion (World Health Organization [WHO], 2020). Iran is also facing an increase in the number of older adults, and according to UN statistics in 2006, the number of people over 60 years of age in Iran accounted for 6% of the total population, i.e., a population of 4,562,000. According to forecasts, this figure will reach 263,930,000 people by 2050, which is a population equivalent to 26% of the total population (Mehri et al., 2020).

Aging is a phenomenon that needs to be managed because older people often experience changes that can sometimes lead to physical, mental, and social limitations that overshadow their quality of life (Samarakoon et al., 2011; McPhee et al., 2016). Self-care, as a health-promoting behavior, can help older people manage the consequences of these changes (Callaghan, 2005). Self-care must be planned to meet all the needs of old people (Goes et al., 2020). Self-regulation is a mechanism that can help implement effective self-care (Koch and Nafziger, 2011). It is a goal-oriented process that increases the capacity for planning to initiate appropriate behavior and control inappropriate behavior (Mann et al., 2013; Wehmeyer and Shogren, 2017).

There is a controversy among scientists about self-regulatory steps. Bandura proposed that self-regulation consists of three steps: self-observation, judgment, and self-response (Bandura and Jourden, 1991). Kanfer proposed a three-step theory that includes self-monitoring, self-evaluation, and self-reinforcement (Kanfer, 1970). Miller and Brown defined self-regulation in seven dimensions, which include information input, self-monitoring, triggering change, searching for options, planning, implementation, and assessing the plan’s effectiveness (Miller and Brown, 1991).

Because measuring the level of self-regulation in older adults is necessary for self-care planning, it is beneficial to use an appropriate questionnaire based on the self-regulation steps and tailor it to the older population. According to our search, several questionnaires, such as the Beaufort Self-Regulatory Questionnaire and the Barnard Self-Regulatory Learning Questionnaire, measured only one aspect of self-regulation, namely self-regulated learning (Taghizade et al., 2020; Tahmasbipour et al., 2021). Fortunately, one of the oldest and most common questionnaires in accordance with our goal was the 31-item Short Form Self-Regulatory Questionnaire (SSRQ), which is derived from the 63-item Self-Regulatory Questionnaire (SRQ; Brown et al., 1999). This questionnaire can measure older adults’ general ability to regulate behavior and has been extensively validated across different populations and cultures (Garzon Umerenkova et al., 2017; Chen and Lin, 2018). Carey et al. extracted the SSRQ from the SRQ and considered one dimension that indicated overall self-regulatory capacity (Carey et al., 2004). In 2005, Neil and Carey assessed the psychometric properties of the SSRQ, and two dimensions, including impulse control and goal setting, were introduced through factor analysis (Neal and Carey, 2005). In 2009, Potgieter and Botha conducted a study on students and identified seven factors: monitoring, decision-making, learning from mistakes, mindful awareness, perseverance, creativity, and self-evaluation (Potgieter and Botha, 2009).

It has been suggested that the dimensions of self-regulation may differ among population groups and different cultures (Garzon Umerenkova et al., 2017; Chen and Lin, 2018). Therefore, it was necessary to conduct an independent and purposeful study for the psychometrics of this questionnaire in Iran. Zeinali et al. (2011), in the section “Materials and Methods” of one study in several lines, briefly reported the psychometric properties of the SSRQ for use in Iranian adolescents and proposed a 28-item version. As the participants in our study were older adults, this tool had to be tailored to their culture and abilities. According to our search, the psychometric properties of SSRQ for measuring self-regulation in Iranian older adults had not been evaluated. In addition, no cutoff point was reported in various versions. The evidence indicated that determining the cutoff point is important because it acts as a classification boundary and provides a boundary for interpreting scores above and below that point (Carle et al., 2011). Therefore, the main objectives of this study were to assess the psychometric properties of the SSRQ in Iranian older adults, identify subscales based on the constructs of self-regulation theory, and finally determine optimal cutoff point.

## Materials and Methods

This cross-sectional study was conducted in Shiraz from November 2020 to March 2021, and 500 older adults over the age of 60 were selected using a two-stage convenience sampling method (stage 1, *n* = 250; stage 2, *n* = 250). Due to the lack of access to the initial participants, the use of two samples prevented the effect of being familiar with the first questionnaire when completing the second questionnaire and as a better questionnaire response after reducing items. Community-dwelling older adults were recruited using two types of convenience sampling techniques including grab approach and snowball sampling. Due to the epidemic, in a grab sampling approach, we visited nursing homes and urban health centers and, after checking medical records, contacted eligible older adults, and invited them to participate in the study and fill out an online questionnaire *via* WhatsApp or a link. We used the snowball technique to send questionnaires *via* WhatsApp to older people who were introduced by their peers after testing their cognitive status with questions from the researcher. The inclusion criteria invovled community-dwelling adults aged 60 and older with at least an elementary level of literacy, a smartphone, and internet access. The exclusion criteria included older adults who had persistent severe psychological problems and were reluctant to participate in the study.

### Tools

#### Short Form Self-Regulation Questionnaire

We used the questionnaire developed by Carey et al. (2004) to assess self-regulation behavior in older adults. This self-reported questionnaire contains 31 items, each item was scored on a five-point Likert scale ranging from 1 (strongly disagree) to 5 (strongly agree). The questionnaire scores ranged from 31 to 155, with higher scores indicating better self-regulation behavior.

#### Generalized Self-Efficacy Scale

The Generalized Self-Efficacy Scale (GSE-10) is a 10-item scale developed by Schwarzer and Jerusalem in 1979. It was rated on a four-point Likert scale ranging from not at all true = 1 to completely true = 4. The total self-efficacy score was obtained by summing the item score and ranges from 10 to 40 (Singh et al., 2009). This scale was translated into Persian in 1996 (Nezami et al., 1996), then Rajabi and Moeini et al. verified its validity and reliability and reported Cronbach’s α of the scale as 0.82 and 0.81, respectively (Rajabi, 2006; Moeini et al., 2008). Moreover, the present study obtained a Cronbach’s α of 0.75 for the Persian version of GSE-10 among older adults.

### Procedure

First, permission was obtained from the original questionnaire’s developer (Dr. Kate B Carey affiliated with Brown University School of Public Health, Providence, United States). This research was then divided into two stages: the first stage included tool translation technique and cultural adaptation. The second stage involved evaluating the psychometric properties of the tool to examine its validity (face, content, construct, and convergent validity). In the first step, the SSRQ was translated into Persian using the standard forward–backward technique (Wild et al., 2005). To determine face validity, SSRQ was completed through interviews with 10 subjects to ensure linguistic and conceptual equivalence of translations. Based on the opinions of the research team, the tool was modified and the final questionnaire was created. To calculate the qualitative content validity, 10 health psychologists who were familiar with the psychometric process were asked to comment on the position and the grammar of the items, and the use of appropriate words in the phrases. In addition, the content validity ratio (CVR) and the content validity index (CVI) were examined to calculate the quantitative content validity of the questionnaire. To determine CVR, 10 experts (in the fields of health education, psychology, nursing, public health, and gerontology who were familiar with the subject matter) were asked independently to rate items using a three-point ranking scale (necessary, helpful but unnecessary, and unnecessary). According to the Lawshe table, the minimum agreed CVR value based on evaluations of 10 experts should be greater than 0.62 (Lawshe, 1975). Finally, the mean CVR value of all SSRQ items was determined to be 0.84, and the CVR value of each questionnaire item was higher than the minimum acceptable range.

The CVI score of each item was calculated using Waltz and Bausell’s method (Waltz and Bausell, 1981). Therefore, experts were asked to determine the degree of relevance, clarity, and simplicity of each item using a four-part spectrum. Then, the number of experts who chose options three and four was divided by the total number of experts. Lastly, the CVI of SSRQ was calculated using the mean of the CVI scores for the entire item (0.90).

To assess construct validity, in the first step with 250 participants, the kurtosis test and data normality were determined (Garson, 2012). Then, the SSRQ factor structure was determined using exploratory factor analysis (EFA) through SPSS version 23. The Kaiser–Meyer–Olkin (KMO) test and Bartlett’s test for sphericity were used to determine sampling adequacy and the appropriateness of the factor analysis. Then, principal component analysis in promax rotation was performed to extract latent factors and appropriate items from the factors. Each item was assigned to a factor based on communalities greater than 0.3 (Samuels, 2017). In the next step, the confirmatory factor analysis (CFA) was performed on the second sample data (*n* = 250) using structural equation modeling with AMOS 24. Therefore, first- and second-order models were designed, and fit indices based on cutoff values were reported.

Convergent validity is one of the issues related to construct validity and a study that tests with similar constructs should have an acceptable correlation (Hopkins, 2017). To acquire convergent validity, Pearson’s correlation was used between the components of the SSRQ and the total score of GSE-10. Reliability was examined based on the Cronbach’s α coefficient, and the receiver operating characteristic (ROC) curve was performed to estimate the optimal cutoff point using SPSS version 23. It is worth noting that SPSS software calculates the area under the curve (AUC) value, sensitivity, specificity, *p*-value, and confidence interval as well as Youden’s J, K-index, and DIFF using the formula.

## Results

### Participants

A total of 500 older people participated in the study in two stages. Most of the older adults were women (*n* = 307; 61.4%), were married (*n* = 408; 81%), were between the ages of 60 and 70 years (*n* = 402; 80.4%), and had a high school diploma or less (*n* = 435; 87%). The majority of participants reported that they were living with family (*n* = 434; 86.8%). Table 1 presents the characteristics of the study population in samples 1 and 2.

**TABLE 1:** Demographic characteristics of the older adults in samples 1 and 2.

	Sample 1 (*n* = 250)	Sample 2 (*n* = 250)
	*N* (%)	*N* (%)
***Gender***		
Men	98 (39.2)	95 (38)
Women	152 (60.8)	155(62)
***Age***		
60–70	189 (75.6)	213 (85.2)
70–80	49 (19.6)	31 (12.4)
80^+^	12 (4.8)	6 (2.4)
***Marital status***		
Single	25 (10)	11 (4.4)
Married	198 (79.2)	210 (84)
Divorced/Separated/Widow	27 (10.8)	29 (11.6)
***Education***		
High school grade(diploma) or less	209 (83.6)	226 (84.4)
Academic education	41 (16.4)	54 (15.6)
***Medical history***		
Healthy	91 (36.4)	108 (43.2)
Diabetes	80 (32)	70 (28)
Hypertension	55 (22)	44 (17.6)
Depression	12 (4.8)	9 (3.6)
Other	12 (4.8)	19 (7.6)
***Living arrangement***		
With family or relatives	203 (81.2)	231 (92.4)
Living alone	47 (18.8)	19 (7.6)

### Construct Validity

Kurtosis values for 3, 8, 11, 16, and 31 items in the first sample were −1.12, −1.11, −1.16, −1.09, and −1.09, respectively, so these items were omitted. A normality test was done, and five outlier participants were excluded from the analysis based on the boxplot (with numbers: 49, 51, 68, 86, and 88).

#### Results of Exploratory Factor Analysis

Good results of Bartlett’s test of sphericity (χ^2^ = 2,358.792; *p* < 0.001) and the KMO test (KMO = 0.791) showed sampling adequacy and provided minimum standards for conducting a factor analysis (Kaiser, 1974; Field, 2013). Therefore, the 26-item questionnaire was subjected to principal component analysis estimation using the promax rotation. Here, six items (1–6–9–15–23–26) were removed due to absolute value < 0.3, and finally, 20 items were left. EFA extracted four factors. Using the self-regulatory theory as a reference framework, a panel of experts from the fields of health promotion, gerontology, and psychology named four factors. They were categorized as follows: self-awareness (six items), goal setting (two items), action planning (six items), and self-monitoring (six items; see Table 2).

**TABLE 2:** The four factors of the Short Form Self-Regulatory Questionnaire (SSRQ) in the Iranian older adults and their factor loadings (*n* = 250).

20-Items		Factor loading			
		Self-awareness	Goal setting	Action planning	Self-monitoring
Q2	I have trouble making up my mind about things.	0.778			
Q4	I don’t notice the effects of my actions until it is too late.	0.852			
Q5	I am able to accomplish goals I set for myself.		0.833		
Q7	It’s hard for me to notice when I’ve “had enough” (alcohol, food, sweets).				0.845
Q10	I have trouble following through with things once I’ve made up my mind to do something.			0.693	
Q12	I can stick to a plan that’s working well.			0.661	
Q13	I usually only have to make a mistake one time in order to learn from it.				0.805
Q14	I have personal standards, and try to live up to them.				0.523
Q17	I have a lot of willpower				0.342
Q18	When I’m trying to change something, I pay a lot of attention to how I’m doing.			0.521	
Q19	I have trouble making plans to help me reach my goals.			0.775	
Q20	I am able to resist temptation.	0.746			
Q21	I set goals for myself and keep track of my progress.		0.316		
Q22	Most of the time I don’t pay attention to what I’m doing.	0.832			
Q24	I can usually find several different possibilities when I want to change something.			0.705	
Q25	Once I have a goal, I can usually plan how to reach it			0.570	
Q27	Often, I don’t notice what I’m doing until someone calls it to my attention.	0.800			
Q28	I usually think before I act				0.339
Q29	I learn from my mistakes	0.734			
Q30	I know how I want to be				0.320
*Total variance:* 76.34%					

#### Results of Confirmatory Factor Analysis

**FIGURE 1:**
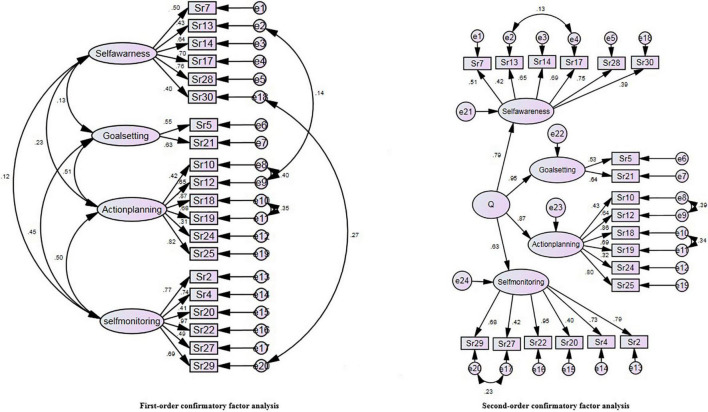
Modified first- and second-order models.

In this step, the EFA result was confirmed by performing CFA on the second sample (*n* = 250). The first- and second-order CFA models were modified using AMOS software proposed correction command, and satisfactory fit indices were found. Table 3 presents a comparison of the fit indices of second-order CFA to the first-order model, and Figure 1 shows the path analysis of modified first- and second-order models.

**TABLE 3:** Fit indices for the SSRQ among older adults.

Indexes	χ2/df	Sig.	RMSEA	CFI	GFI	IFI	PRATIO	PCFI
First-order model	3.41	<0.001	0.08	0.79	0.82	0.79	0.86	0.68
Modified first-order model	1.87	0.07	0.05	0.92	0.89	0.92	0.84	0.78
Second-order model	3.62	<0.001	0.09	0.80	0.80	0.75	0.85	0.64
Modified second-order model	2.05	0.05	0.06	0.90	0.86	0.90	0.80	0.77

#### Convergent Validity

The results of Pearson’s correlation demonstrated a significant positive correlation between the SSRQ-20 and its subscale scores and the GSE-10 (*p* < 0.01). These findings demonstrated acceptable convergent validity. The interscale correlation between SSRQ-20 subscales was also significantly positive, which further confirmed the construct validity (Table 4).

**TABLE 4:** Pearson’s correlation between the components of SSRQ and the Generalized Self-Efficacy Scale (GSE-10) in older adults.

	Mean (SD)	1	2	3	4	5	6
1. GSE-10	32.28(3.69)	1					
2. SSRQ, Self-awareness	21.32(3.78)	0.38**	1				
3. SSRQ, Goal setting	7.83(1.75)	0.32**	0.58**	1			
4. SSRQ, Action planning	22.99(4.03)	0.44**	0.68**	0.70**	1		
5. SSRQ, Self-monitoring	21.24(3.73)	0.34**	0.63**	0.67**	0.70**	1	
6. SSRQ, Total	73.40(11.54)	0.44**	0.85**	0.80**	0.90**	0.88**	1

**p ≤ 0.01.

### Reliability Analysis

The reliability of the 31-item SSRQ and our final 20-item scale in older adults was estimated to be 0.82 and 0.87, respectively, *via* Cronbach’s α coefficients.

### Scoring, Receiver Operating Characteristic Curve, and Cut-Off Points

**FIGURE 2:**
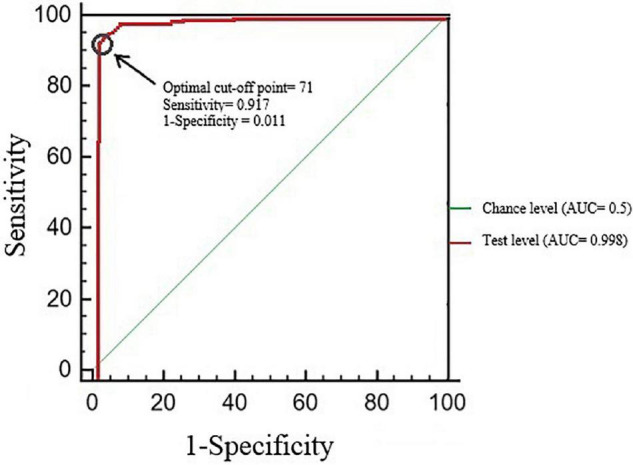
Receiver operating characteristic (ROC) curve.

The total score represented the sum of the points of each item. The item scores ranged between 1 and 5; thus, the total scores ranged from 20 to 100. The cutoff point of SSRQ-20 was determined using the ROC curve distribution. The mode score of the 20-item scale (mode = 71) was assigned as the cutoff point due to an excellent AUC (Figure 2), high sensitivity, and high specificity. Youden’s J index (J) is a coefficient that maximizes the sensitivity and specificity of the cutoff point (Perkins and Schisterman, 2006). As Youden’s J index (J) was ≥ 0.60 and DIFF ≤ 0.2, the specified cutoff point of SSRQ-20 is optimal (Table 5). In the second sample, 157 (62.8%) participants scored higher than 71.

**TABLE 5:** AUC value, sensitivity, and specificity of ROC curve for 20-item SSRQ.

Scale	AUC	95% CI		*P*-value	Cut-off Point	Sensitivity	1-Specificity	Youden’s J	Distance Sqrt. (K-Index)	DIFF
		Lower Bound	Upper Bound							
SSRQ	0.998	0.995	1	0.000	71	0.917	0.011	0.993	0.002	0.003

*p ≤ 0.05; AUC = area under curve; CI = confidence interval; DIFF = abs (sensitivity– specificity); D Value or K-Index = Sqrt [(1 - Sensitivity)^2^ + (1 - Specificity)^2^; Kallner, 2018)].*

## Discussion

The three main objectives of this study were to assess the validity and reliability of the SSRQ in an Iranian older adult population sample and to identify subscales and cutoff points. We used precise methods based on psychometric criteria to confirm the validity and reliability of the Persian version of the SSRQ, and we obtained a questionnaire with four subscales and 20 items. With proper fit indices, the final SSRQ was shorter than the original. Similar to our study, Chen and Lin found that, while validating the SSRQ in Taiwanese students, the number of questionnaire items was reduced to 22 items and a shorter questionnaire was obtained (Chen and Lin, 2018). It seems that a decrease in the number of questionnaire items compared to the original one may be related to cultural, environmental, and population group differences. On the other hand, we believe that the shortness of the questionnaire is a good feature of a measure for use in the older population because it is much faster to complete and more practical due to vision dysfunction and reading problems of older people. Bowling et al. proposed that the use of short scales in older adults can improve measurement accuracy (Feizi and Heidari, 2020).

In this study, we obtained four subscales for the SSRQ in Iranian older population including self-awareness, goal setting, action planning, and self-monitoring. The subscales were named based on the content of their subitems and using the constructs of the self-regulation theory. These subscales are somewhat consistent with the Bandura self-regulatory stages (Bandura and Jourden, 1991). Bandura proposed that self-observation as the first step of self-regulation is a process that involves the awareness of thoughts and feelings to determine the goal (Bandura, 1991). Therefore, self-observation is nearly synonymous with self-awareness and goal-setting sub-scales. In our study, action planning is a unique concept that is defined as a stage after goal setting to create behavior (Ogden, 2019). Action planning is the process of transforming people’s strategies and goals into action (Gagné, 2018). In Bandura’s theory, judgment and self-response are also consistent with the self-monitoring subscale. Self-monitoring is a behavior change technique that includes the ability to monitor and regulate one’s emotions and behaviors in response to changes and problems (Bruhn et al., 2015). According to the evidence, other researchers have identified a variety of dimensions for SSRQ (Neal and Carey, 2005; Potgieter and Botha, 2009). This could be due to the diversity of researchers’ viewpoints and participants responses.

Regarding convergent validity, the GSE-10 for Iranian older adults demonstrated a positive and significant correlation with SSRQ total score and subscales, which displays the expected convergent validity. Convergent validity was not used in previous similar studies (Potgieter and Botha, 2009; Garzon Umerenkova et al., 2017; Chen and Lin, 2018). The reason for using the GSE-10 for convergent validity in our study was that it is a short scale that can be filled out more easily by old people and also is developed to measure self-beliefs to meet a variety of difficult situations (Lazić et al., 2021). Therefore, the contents of both scales pursue almost the same goal, which is to measure self-efficacy and self-regulation as Bandura’s significant constructs for predicting behavior to improve self-care (Luszczynska et al., 2005; Pillay et al., 2022).

According to the results, the “mode” score is an optimal cutoff point for the SSRQ-20. Therefore, people who scored higher than 71 have better self-regulatory behavior, and those who scored below indicate poor self-regulatiory behavior. It should be noted that the cutting point of the SSRQ has not been identified in previous studies, hence this is one of the highlights of the present study.

### Strengths and Limitations

The strengths of this study include the adaptation and validation of the Persian version of the SSRQ in older people, the use of separate samples for EFA and CFA, and the development of a cutoff point for the scale. There are several limitations in this study. The sample size might be relatively small, and we clinically assessed the cognitive status in older people using several questions and without using objective tools. In addition, the Iranian population consists of different ethnic groups, and the present study was conducted on the Fars ethnicity, which is the largest ethnicity in Iran. Therefore, caution should be exercised when generalizing the findings to other ethnic minorities, such as Kurds, Turkmen, and Baloch. On the other hand, this study was conducted during the COVID-19 outbreak; therefore, it took longer to fill out the questionnaires than expected. Also, as the questionnaires were sent electronically or *via* WhatsApp to the participants, it was not possible to reach the poor, illiterate, marginalized, rural people, and those without smartphones.

## Conclusion

In the Iranian context, the short version of the SSRQ with 20 items demonstrated acceptable psychometric properties and a good factor structure for measuring self-regulation in older adults. Due to features such as good reliability and validity, the design of subscales based on the self-regulatory theory, on filling of the forms in a short period of time by older adults, and the determination of the cutoff point, it seems that the SSRQ-20 is a suitable tool for planners to use in designing interventions aimed at improving self-regulation in older adults.

## Data Availability Statement

The original contributions presented in this study are included in the article, further inquiries can be directed to the corresponding author.

## Ethics Statement

The studies involving human participants were reviewed and approved by Ethics Committee of Shiraz University of Medical Science (Ref. no: IR.SUMS.REC.1398.1365). The patients/participants provided their written informed consent to participate in this study.

## Author Contributions

MK conceptualized the study project, supervised the implementation process, and edited the manuscript. MM-J participated in study development, data collection, data analysis, and wrote the manuscript. AM contributed to data collection and edited the manuscript. AA helped in data analysis and edited the manuscript. All authors have read and approved the final manuscript.

## Conflict of Interest

The authors declare that the research was conducted in the absence of any commercial or financial relationships that could be construed as a potential conflict of interest.

## Publisher’s Note

All claims expressed in this article are solely those of the authors and do not necessarily represent those of their affiliated organizations, or those of the publisher, the editors and the reviewers. Any product that may be evaluated in this article, or claim that may be made by its manufacturer, is not guaranteed or endorsed by the publisher.
